# Wellens’ Sign and Syndrome Detected With the Home Use of a Novel 12-Lead Electrocardiogram

**DOI:** 10.1016/j.jaccas.2026.109186

**Published:** 2026-07-29

**Authors:** David E. Albert, Stavros Stavrakis

**Affiliations:** a AliveCor, Inc, Mountain View, California, USA; b University of Oklahoma Health Center, Oklahoma City, Oklahoma, USA

**Keywords:** Kardia12L, myocardial infarction, Wellens’ sign, Wellens’ syndrome

## Abstract

**Background:**

Wellens’ sign is a high-risk electrocardiogram (ECG) pattern associated with proximal left anterior descending artery stenoses and high risk of major adverse cardiovascular events.

**Early Reports Summary:**

A man with transient chest pain remained asymptomatic including with exertion for 48 hours. His friend performs a home 12-lead ECG using a novel, artificial intelligence–enabled device that showed a classic Wellens’ Sign Type A, and the man went to a hospital and subsequently received 2 stents in his proximal left anterior descending artery.

**Discussion:**

The Kardia12L is a Food and Drug Administration–cleared, pocket-sized ECG device that generates an interpreted 12-lead ECG. Devices like Kardia 12L can lead to diagnosis of cardiovascular disease from home and improve outcomes.

**Novelty:**

This case highlights the utilization of novel technology to facilitate accurate diagnosis.

**Take-Home Messages:**

This case illustrates Wellens’ Type A Sign and Syndrome in an asymptomatic patient with a history of chest pain. New technology enables the recording of a diagnostic ECG in high-risk patients anywhere.

Wellens’ syndrome is a high-risk electrocardiographic (ECG) pattern associated with critical proximal left anterior descending artery disease and impending anterior myocardial infarction.^1^ First described by Hein Wellens, it is characterized by anterior T-wave abnormalities that often appear when patients are asymptomatic, making recognition essential to avoid delayed diagnosis and adverse outcomes. Advances in artificial intelligence (AI) have enabled accurate ECG interpretation using reduced-lead systems. The Kardia 12L device (AliveCor) is a portable, Food and Drug Administration (FDA)-cleared platform that reconstructs a 12-lead ECG from limited electrodes using deep learning algorithms. We present a case of asymptomatic Wellens’ syndrome identified in a nonclinical setting using an AI-enabled reduced-lead ECG, highlighting the importance of early recognition and the potential of emerging technologies to facilitate timely diagnosis.

**Take-Home Messages**

• Be able to recognize a Wellens’ Type A Sign and Syndrome in an asymptomatic patient with a history of chest pain and understand the need for interventional therapy to improve outcomes.
• Be aware of new technology enabling the recording of a diagnostic ECG in many nonmedical environments and the potential to detect high-risk patients anywhere.

## Case Summary

**Figure 1:**
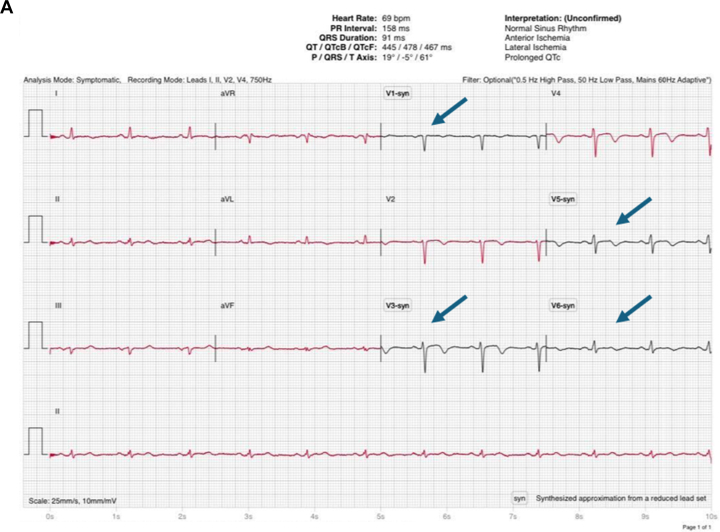

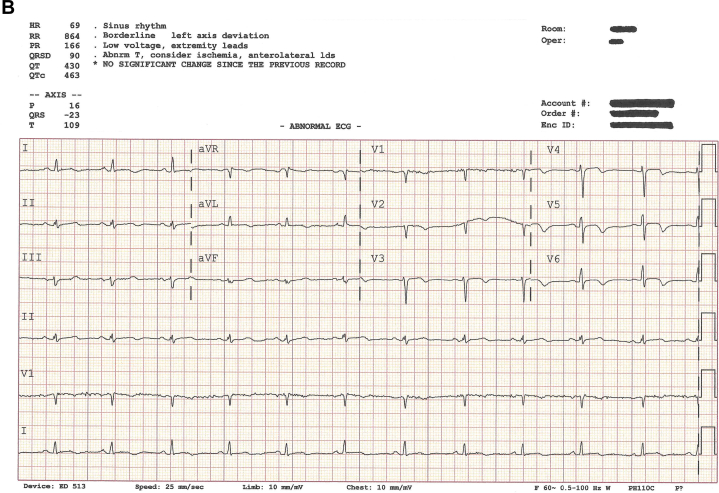
AI Synthesized 12-Lead ECG and Standard 12-Lead ECG (A) Kardia 12L novel 12-lead electrocardiogram (ECG) recorded by a friend with no prior ECG experience at home on the asymptomatic subject. Blue arrows represent the artificial intelligence–reconstructed leads. (B) Standard 12-lead ECG. Standard ECG taken in the emergency department.

**Figure 2:**
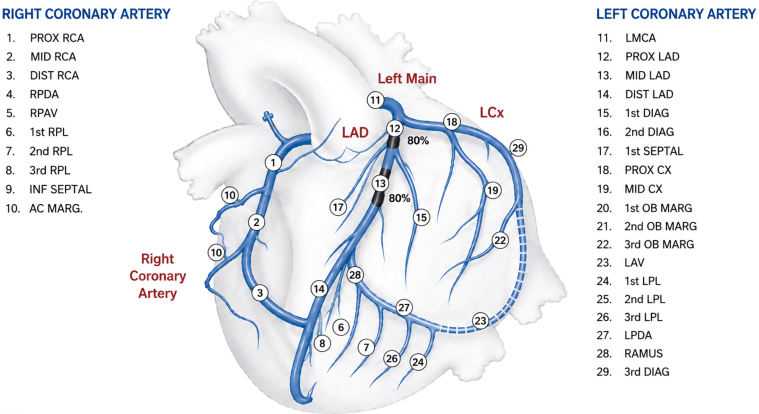
Cardiac Catheterization Report Proximal 80% left anterior descending artery (LAD) and mid-LAD stenoses that were both stented. LCx = left circumflex artery.

A 57-year-old man with no previous cardiac history was at a gym for his normal exercise routine and suddenly felt severe chest discomfort and thought he was “having a heart attack.” He stopped exercising and rested for 15 minutes, and the chest discomfort went away. He went home and told his wife, who was not impressed with his story. He had no more symptoms. Two days later, he went on a 7-mile hike, remained asymptomatic, but told the story of his chest pain at the gym to his friend on the hike who happened to be an AliveCor senior engineer. That engineer went to the office and brought a prototype reduced-lead, Deep Neural Network–enabled 12-lead ECG, the Kardia12L to the man’s house and performed a recording. It is of note that this engineer had never performed a 12-lead ECG before but had seen the first author perform several of them and explain where to place the 5 electrodes. Once completing the recording (Figure 1A), the engineer transmitted the ECG to the first author for review and recalled the story of the severe chest pain that resolved. It was 8 pm on a Saturday night the day before a holiday (St Patrick’s Day). Despite the timing, the first author told the engineer to take his friend to an emergency department (ED) of a major hospital immediately. Given that the man had been asymptomatic for 48 hours even with exertion, he was reluctant to go to the ED, but he was eventually convinced. On arrival at the ED, his 12-lead ECG was recorded (Figure 1B), which was identical to the home Kardia12L recording in both appearance and algorithmic interpretation, showing antero-lateral ischemia. The emergency physician had a high-sensitivity troponin measured and it came back significantly elevated at 2,665 ng/L. Given the lack of symptoms over the preceding 48 hours, but with the ECG and high-sensitivity troponin data, the man was admitted to the coronary care unit for observation. The next day, he was taken to the cardiac catheterization lab and found to have an 80% stenosis of his proximal left anterior descending artery before any branch and another 80% stenosis in the mid left anterior descending artery. He received 2 drug-eluting stents and was discharged home. (Figure 2). Both the Kardia12L and the conventional 12-lead ECG gave algorithmic interpretations of antero-lateral ischemia. The patient was asymptomatic and had gone on a 7-mile hike with no symptoms earlier that evening so ongoing ischemia seemed unlikely. The patient has remained asymptomatic over the past year and resumed his regular exercise routine.

## Novelty of the Submission

Although Wellens’ sign has been previously reported, this case highlights the utilization of a novel technology with AI to help accurate diagnosis and treatment.

## Future Directions

**Figure 3:**
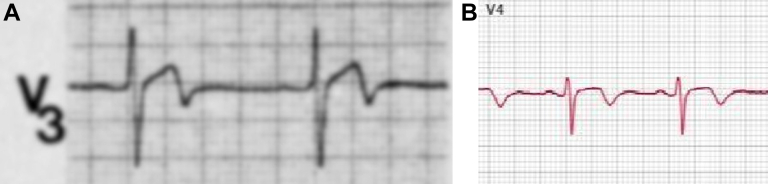
Wellens’ Biphasic T-wave in Precordial Leads (A) V_2_ ECG. V_2_ ECG showing Wellen’s sign Type A from the original 1982 paper, reproduced with permission. This morphology manifests only at rest in the anterior precordial leads. (B) V_4_ ECG from Kardia12L. Classic biphasic T-wave of Wellen’s Type A that is very similar to Figure 2A. Abbreviation as in Figure 1.

This is a classic story for both Wellens’ Sign Type A and Wellens’ Syndrome. The presentation of a biphasic T-wave in the anterior precordial leads in an asymptomatic patient with a history of previous chest pain was first described in the 1982 paper from Hein Wellens’ group as a marker of a patient at high risk of a large myocardial infarction or sudden death (Figure 3A).^1^ This same pattern was seen in the measured V_2_ and V_4_ of the Kardia 12L (Figure 3B). This ECG sign disappears in the presence of chest pain. Subsequent papers and case reports have proven that this sign and its type B presentation are major risk factors for an anterior myocardial infarction or sudden death and are considered by the guidelines as a reperfused ST-segment elevation myocardial infarction (STEMI) equivalent needing immediate intervention in the form of stenting or coronary bypass surgery.^2^, ^3^, ^4^, ^5^ Of note, type B (deeply and symmetrically inverted T waves) is more frequent than type A (biphasic T waves, with initial positivity and terminal negativity). In both types, the ECG criteria for Wellens' syndrome exclude pathologic Q waves, left or right bundle branch block, left or right ventricular hypertrophy, and poor R wave progression. In addition, this case highlights the importance of serial ECGs in the setting of chest pain, as the initial ECG may appear normal.

**Figure 4:**
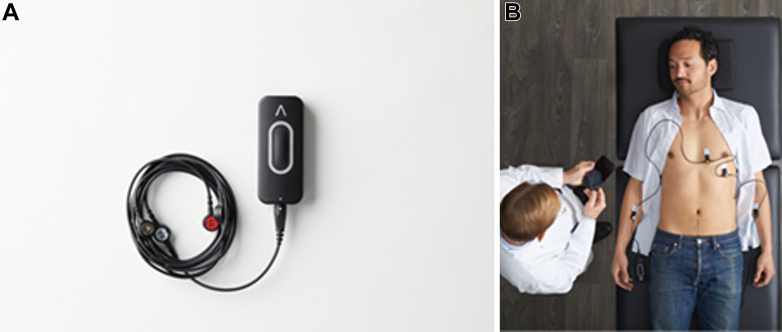
Kardia 12L AI-Driven ECG and Kardia 12L on Patient With 5 Electrodes (A) Kardia12L. Artificial intelligence (AI)-powered, reduced lead with single electrode cable design. The pocketable pod communicates via Bluetooth Low Energy with a smartphone or tablet to record, interpret, and transmit the 12-lead ECG. (B) Kardia12L on patient. Novel 5-electrode cable design providing diagnostic 12-lead ECG that is simple to use, pocketable in size, and powered by AI trained on millions of ECGs. Abbreviation as in Figure 1.

The Kardia12L is a recently FDA-cleared,^6^ pocket-sized and smart device–powered (smartphone or tablet) ECG device using only 5 electrodes (I, II, V_2_, V_4_) to generate an interpreted 12-lead ECG using AI trained on millions of ECGs.^7^ To reconstruct a full 12-lead ECG from a subset (leads I, II, V_2_, and V_4_), Kardia 12L used a machine-learning algorithm trained on a dataset of 1,000,000 standard 12-lead recordings. The model was optimized to minimize the error between the synthesized leads (V_1_, V_3_, V_5_, and V_6_) and the original gold-standard measurements, ensuring high waveform fidelity. The primary advantage of this AI-driven approach is its ability to interpret ECGs where standard clinical rules do not yet exist. Although conventional algorithms are “feature-dependent”—meaning, they can fail if a single wave is mismeasured—our model learns to recognize the global context of the cardiac morphologies and rhythms. This allows for accurate interpretation based on leads I, II, V_1_/V_2_, and V_4_ alone, effectively “filling the gap” where traditional diagnostic criteria are unavailable for reduced-lead systems. This report highlights the notion that Kardia 12L is very simple to use by people with little or no training, potentially enabling the identification of high-risk individuals in almost any location (Figures 4A and 4B). Of note, Kardia 12L is the first reduced-lead cardiograph that is FDA-cleared to provide the diagnosis of acute myocardial infarction.^6^ It is available for purchase by health care professionals. Although the cost-effectiveness has not been systematically evaluated, it costs about half the price of a traditional 12-lead ECG machine.

Great disparities concerning racial and ethnic differences in relation to cardiovascular disease in the United States continue to persist, driven by social determinants of health, clinical factors, and lifestyle behaviors.^8^^,^^9^ Increasing use and acceptability of devices like Kardia 12L, leading to diagnosis of cardiovascular disease from one’s home or any venue, could increase health equity in the hands of those most affected by cardiovascular disparities.

## Conclusions

Many locations across the globe have no access to conventional 12-lead ECG equipment or expert interpretation. The rapid identification of STEMI and STEMI-equivalent patients to facilitate acute percutaneous coronary intervention is clearly needed. This case demonstrates the potential of one such technology.

## Funding Support and Author Disclosures

Dr Albert is the founder and chief medical officer of AliveCor; is a member of the board of directors; has received significant salary from AliveCor; and is a significant equity holder in AliveCor. Dr Stavrakis has reported that he has no relationships relevant to the contents of this paper to disclose.
